# Cytokines, Fatigue, and Cutaneous Erythema in Early Stage Breast Cancer Patients Receiving Adjuvant Radiation Therapy

**DOI:** 10.1155/2014/523568

**Published:** 2014-03-31

**Authors:** Vitaliana De Sanctis, Linda Agolli, Vincenzo Visco, Flavia Monaco, Roberta Muni, Alessandra Spagnoli, Barbara Campanella, Maurizio Valeriani, Giuseppe Minniti, Mattia F. Osti, Claudio Amanti, Patrizia Pellegrini, Serena Brunetti, Anna Costantini, Marco Alfò, Maria Rosaria Torrisi, Paolo Marchetti, Riccardo Maurizi Enrici

**Affiliations:** ^1^Department of Translational Medicine, Institute of Radiation Oncology, “Sapienza” University, Sant'Andrea Hospital, Via di Grottarossa 1035-1039, 00189 Rome, Italy; ^2^Department of Clinical and Molecular Medicine, Cellular Diagnostic Unit, “Sapienza” University, Sant'Andrea Hospital, Via di Grottarossa 1035, 00189 Rome, Italy; ^3^Statistics, Probability and Applied Statistics, “Sapienza” University, Piazzale Aldo Moro 5, 00185 Rome, Italy; ^4^Department of Translational Medicine, Breast Surgery, “Sapienza” University, Sant'Andrea Hospital, Via di Grottarossa 1035, 00189 Rome, Italy; ^5^Department of Clinical and Molecular Medicine, Medical Oncology, “Sapienza” University and IDI-IRCCS, Sant'Andrea Hospital, Via di Grottarossa 1035, 00189 Rome, Italy; ^6^UOD Psyco-oncology, Sant'Andrea Hospital, Via di Grottarossa 1035, 00189 Rome, Italy

## Abstract

We investigated the hypothesis that patients developing high-grade erythema of the breast skin during radiation treatment could be more likely to present increased levels of proinflammatory cytokines which may lead, in turn, to associated fatigue. Forty women with early stage breast cancer who received adjuvant radiotherapy were enrolled from 2007 to 2010. Fatigue symptoms, erythema, and cytokine levels (IL-1**β**, IL-2, IL6, IL-8, TNF-**α**, and MCP-1) were registered at baseline, during treatment, and after radiotherapy completion. Seven (17.5%) patients presented fatigue without associated depression/anxiety. Grade ≥2 erythema was observed in 5 of these 7 patients. IL-1**β**, IL-2, IL-6, and TNF-**α** were statistically increased 4 weeks after radiotherapy (*P* < 0.05). After the Heckman two-step analysis, a statistically significant influence of skin erythema on proinflammatory markers increase (*P* = 0.00001) was recorded; in the second step, these blood markers showed a significant impact on fatigue (*P* = 0.026). A seeming increase of fatigue, erythema, and proinflammatory markers was observed between the fourth and the fifth week of treatment followed by a decrease after RT. There were no significant effects of hormone therapy, breast volume, and anemia on fatigue. Our study seems to suggest that fatigue is related to high-grade breast skin erythema during radiotherapy through the increase of cytokines levels.

## 1. Introduction

Cancer-related fatigue (CRF) is a common problem among women with breast cancer that receive adjuvant treatment, with a CFR prevalence of 25–99% [1]. Many studies have tried to explain biological mechanisms underlying fatigue during radiotherapy, but no consensus is reached concerning biological bases and potential impact of radiation therapy. Geinitz et al. observed an increase of fatigue during adjuvant radiotherapy (RT) in 40 patients with breast cancer, but they did not find any association with anxiety/depression and/or cytokines levels (TNF-*α*, IL-1*β*, and IL-6) [2]. The study by Noal et al. found no significant association between fatigue and biological disorders in 302 patients undergoing RT and/or adjuvant chemotherapy [3]. Moreover, several studies showed a statistical correlation between persistent fatigue and high levels of proinflammatory markers (IL-1ra, IL-6, and sTNT-RII) in breast cancer survivors [4, 5].

Therefore, factors responsible for fatigue during RT remain still unknown. Among potential causes, the local activation of substances such as systemic inflammatory markers and proinflammatory cytokines has been hypothesized [6–10]. The most frequent acute adverse effect during RT in breast cancer patients is breast skin erythema that has not been evaluated for eventual correlation with fatigue onset [11].

We conducted a prospective study to analyze the potential association between skin erythema and biological blood markers and their influence on radiation-induced fatigue symptoms in patients undergoing RT for early stage breast cancer. The primary hypothesis was that patients developing high-grade erythema of the breast skin during RT could present high levels of serum proinflammatory cytokines and fatigue. A further statistical analysis was to evaluate whether other factors, such as hormonal therapy, breast volume, or anemia, may influence the biological cytokine-related mechanisms leading to fatigue.

## 2. Materials and Methods

This study included early stage breast cancer patients who undergone conservative surgery and RT. Fatigue symptoms, skin erythema, and circulating proinflammatory cytokine levels were registered at baseline, weekly during treatment, at 3 months, and at 6 months from radiotherapy completion. At each assessment, patients completed questionnaires for fatigue and anxiety-depression assessment and provided blood samples for laboratory examinations. Ethics approval was obtained from our institutional review board. All women provided written informed consent.

Only patients who met the following eligibility criteria were included: age ≤75 years, conserving surgery, early stage disease with no nodal involvement (T1N0M0 and TisN0M0), standard postoperative radiotherapy, no previous chemotherapy, lack of significant comorbid conditions (cardiovascular disease, hypertension, respiratory disease, diabetes, cerebral-vascular disease, arthritis, and hypothyroidism). The clinical characteristics of the patients are summarized in Table 1.

The computed tomography-based three-dimensional (3D) planning was employed to determine the clinical target volume (CTV: mammary gland and the above skin).

All patients received external beam radiation therapy delivered by a 6 MV linear accelerator to the CTV. Radiotherapy schedule consisted in a total dose of 50 Gy in 25 daily fractions of 2 Gy each administered 5 days per week. Twenty-one patients (T1-stage) received a subsequent boost to the tumor bed consisting of a total dose of 10 Gy in 4 daily fractions of 2.5 Gy each, delivered with a direct electron field. The breast volume in cubic centimeters (cc) was measured for each patient after treatment planning. We arbitrarily categorized breast volume as large when the treated breast volume was larger than 1000 cc. Hormonal therapy was administered to all stage I patients. Blood samples were taken every week to determine the hemoglobin levels; anemia was considered when hemoglobin <10 gr/dL.

The diagnosis of cancer-related fatigue was made according to the criteria described by Cella et al.: (1) significant fatigue was experienced each day in at least two weeks within the preceding month; (2) the experience of fatigue symptoms resulted in significant distress or impairment of functioning; (3) there is clinical evidence that fatigue is a consequence of cancer or cancer therapy; (4) no concurrent diagnosis of psychiatric disorders (i.e., major depressive disorders) has been made [12–14].

The fatigue severity was identified according to the functional assessment of cancer therapy fatigue subscale (FACT-F), a component of the FACT-G quality of life questionnaire [12–14]. The FACT-F scale includes 13 items (0–5 points for each item); the total score (range: 0–52) corresponds to fatigue severity. According to previous studies, the presence of fatigue was determined by a final score <37 [15, 16]. Subjective symptoms were considered as fatigue symptoms if patients presented a score <37 for two consecutive weeks at FACT-F questionnaires, and no associated depression/anxiety was concurrently present. Anxiety and depression were assessed using the hospital anxiety and depression scale (HADS) [17]. The HADS questionnaire provides a total score ranging from 0 to 21; the threshold for significant clinical levels of anxiety/depression is defined by a total score ≥11. We considered the depression score only, for the purpose of our study.

Treatment related cutaneous toxicity was evaluated according to radiation therapy oncology group morbidity scale [18]. The erythema was defined as high grade when the corresponding grade was ≥2.

Twelve inflammatory cytokines (IL-1*α*, IL-1*β*, IL-2, IL-4, IL-6, IL-8, IL-10, vascular endothelial growth factor (VEGF), epidermal growth factor (EGF), tumor necrosis factor-*α* (TNF-*α*), interferon *γ* (IFN-*γ*), and monocyte chemo-attractant protein-1 (MCP-1)) were tested. For simultaneous and serial assessment of plasmatic cytokines, a multiplex biochip array in Evidence Investigator equipment (Randox Labs. Ltd. Crumlin, UK) was used, as previously reported [19, 20]. Assays were performed on plasma, following the manufacturer's instructions. The analyte's concentration was calculated automatically using routinely generated calibration curves (Evidence Software version 1.4).

The study group consisted of 40 patients (aged from 40 to 73 years) that were managed before, after RT (4 weeks), and during followup (6 months after RT). We achieved a control group including 10 healthy volunteers (aged from 35 to 66 years), whose blood samples were withdrawn once, to define the cutoff value for each cytokine. Plasma was collected and assayed immediately or frozen at −30°C, then avoiding repeated thawing and freezing.

### 2.1. Statistical Methods

To compare the values of proinflammatory markers in healthy donors and for patients during and after the RT, Student's *t*-tests were used. The values are expressed as mean from two independent samples. *P* values < 0.05 were assumed as statistically significant.

Given that we have recorded repeated measurements for each patient, we need to account for dependence in observed fatigue symptoms from the same patient over time. For this purpose, to evaluate the relationship between fatigue and the explanatory variables (erythema, inflammatory markers, hormone therapy, and breast volume) a random effect model has been fitted where individual-specific random effects account for dependence between repeated measures from the same individual. We suppose that fatigue symptoms are related to presence of skin erythema through increased levels of inflammatory markers. However, we should note that corresponding parameter estimates may be severely affected if some of the adopted covariates/factors, for example, in this case increased inflammatory markers, are endogenous, that is, if they are related to random effects and/or random errors in the equation for the presence of fatigue symptoms. In fact, we may think that the presence of skin erythema may lead to increased levels of inflammatory markers and these, in turn, influence the spread of fatigue-related symptoms. At the same time, the increase in levels of proinflammatory markers may be also linked to other covariates that may affect also the presence of fatigue symptoms. Therefore, the link between erythema, proinflammatory markers, and fatigue symptoms could be biased if a proper model is not considered. Since inflammatory markers may be jointly determined with fatigue and, therefore, corresponding estimates in the fatigue equation may be biased by so-called endogeneity bias (for a general treatment of endogeneity bias, see [21]), we decided to correct for this potential endogeneity. Formally, let *i* = 1,…, *n* denote individuals and *t* = 1,…, *T* denote the time occasions the individuals are observed at. The correction method is based on two equations: in the first one, we obtain the residuals from the regression of the endogenous variable (inflammatory markers) on exogenous ones (e.g., skin erythema). This procedure is known as the Heckman two-step correction [22], and it was used to provide more reliable estimates for effects of skin erythema on the increase in inflammatory markers levels, and for effects of the latter on the presence of fatigue-related symptoms. In the first equation, we have

(1)  Markersit∗ =β0+β1Erythemait+bi+εit,Markersit=1⟷Markersit∗>0,bi~N(0,σb2),

where Markers_it_* is a latent Gaussian variable which is binary coded to give the observed inflammatory markers levels Markers_*it*_, indicating whether individual *i* at time *t* presents a level of inflammatory markers beyond a given threshold. The term *β*_0_ represents the overall model intercept and Erythema_*it*_ is an explanatory binary variable indicating whether erythema is present (i.e., any level > 0). The individual specific random effect term, *b*_*i*_, takes into account unobserved sources of heterogeneity between patients, to measure dependence between responses for the same individual; lastly, *ε*_*it*_ is the stochastic error term. This leads to using a generalized linear model with a probit link function [23].

The second equation was used to model the presence of fatigue symptoms (without anxiety) as a function of exogenous (hormone therapy and breast volume) and endogenous (inflammatory markers as a function of skin erythema presence) explanatory variables, plus the residual obtained in the first equation, Mi (Mill's ratio):

(2) Fatigueit∗=γ0+γ1BreastVolumei+γ2HormoneTherapyit+γ3Markersit+γ4Mit+ui+eit,Fatigueit=1⟷Fatigueit∗>0,ui~N(0,σu2),

where, as before, a latent utility Gaussian model has been defined to link the unobserved, true signal for fatigue symptoms (i.e., Fatigue_it_*) to the observed dummy variable describing the presence of fatigue symptoms, Fatigue_*it*_. The term *γ*_0_ represents the overall intercept *γ*_1_, *γ*_2_ and *γ*_3_ represent the effects of explanatory variables, breast volume, hormone therapy (binary), and inflammatory markers (binary), respectively. The term *M*_*it*_ represents the inverse Mills ratio. By definition, it is uncorrelated with exogenous variables that appear in the first probit model and represents the information of inflammatory markers that is not explained by levels of skin erythema. When the parameter *γ*_4_ is significantly different from zero, inflammatory markers may be thought as being jointly determined with fatigue symptoms, and correction is needed.

## 3. Results 

From January 2007 to March 2010, 40 women were consecutively enrolled at our institute.

The mean and median ages for all patients were 54 and 55 years (range: 40–73 years), respectively. Nineteen patients had pathological stage 0 and 21 patients had pathological stage I. Overall, 17 (42.5%) patients have fatigue symptoms arising in the course of radiotherapy and during the followup. On the other hand, depression/anxiety was recorded in 12 (30%) patients at baseline that continue during treatment and followup; 10 patients of them had also fatigue symptoms arising during radiotherapy, while 2 patients did not have fatigue. Therefore, only 7 (17.5%) patients presented fatigue arising in the course of radiotherapy that was not associated to depression/anxiety; only these patients were considered as showing reliable fatigue symptoms. Overall, during radiation therapy, we recorded grade ≥2 erythema in 16/40 (38%) patients; grade ≥2 erythema was observed in 5 of the 7 patients with fatigue symptom.

The plasmatic levels of 12 cytokines were measured in blood samples derived from 40 breast cancer patients before and after RT, during followup, and from 10 healthy donors taken as controls. As expected, some cytokines showed significant differences between healthy donors and pretreated cancer patients (IL-1*β*, IL-2, IL-6, TNF-*α*, and MCP-1). In the whole panel of cytokines, only IL-1*β*, IL-2, IL-6, TNF-*α*, IL-8, and MCP-1 were found to be modified in cancer patients after RT. In particular, IL-1*β*, IL-2, IL-6, and TNF-*α* were significantly increased 4 weeks after RT (*P* < 0.05, compared to pretreated samples), whereas IL-8 and MCP-1 were not significantly altered, as shown in Table 2. The significant increase in cytokine levels was recorded in the 7 patients with fatigue, 5 of them experienced also cutaneous erythema grade ≥2. Nonsignificant modifications have been detected in the other cytokines (data not shown). Interestingly, 6 months after the treatment, only TNF-*α* levels remained significantly higher compared to the pretreated amounts (*P* < 0.05), whereas IL-1*β*, IL-2, and IL-6 were found again not significantly different compared to those observed before RT (See Table 2).

**Figure 1:**
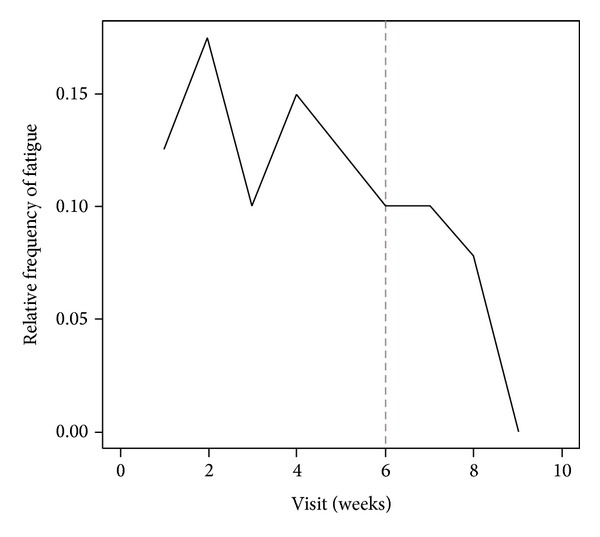
Percentage of patients with fatigue symptoms over time. Dotted line defines the RT completion.

**Figure 2:**
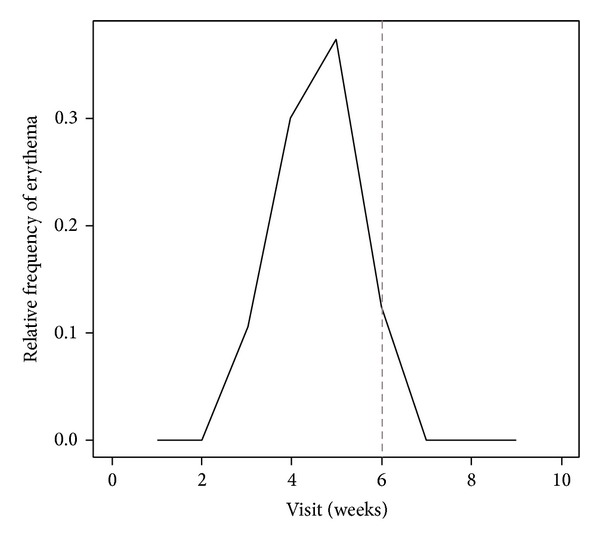
Percentage of patients with skin erythema over time occasions. Dotted line defines the RT completion.

**Figure 3:**
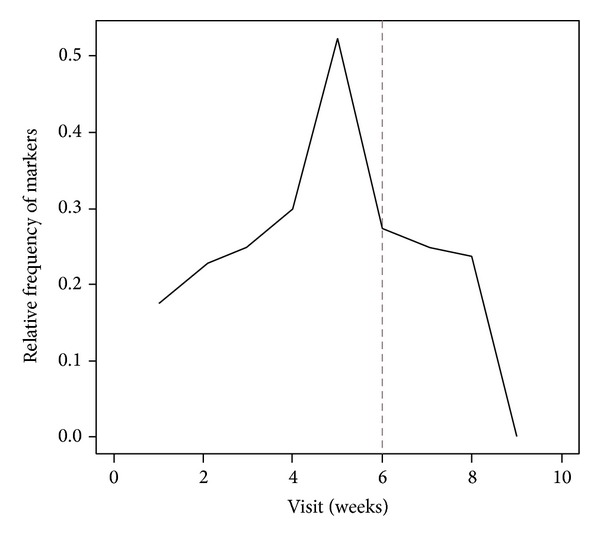
Percentage of patients with increase in inflammatory markers over time occasions. Dotted line defines the RT completion.

After a sudden increase in fatigue prevalence during the early weeks that seems to be unrelated to other factors, results indicated an increase in fatigue from the 3rd week of treatment, peaked between the 4th and the 5th week and decreased after the RT completion (Figure 1). Erythema and proinflammatory marker levels followed fatigue curve during the study, starting to increase in week 3 (Figures 2 and 3). Concomitant peaks on the curves of these three variables were also observed between the fourth and the fifth week of radiation therapy followed by a decrease after RT completion.

The median breast volume in cubic centimeters was 553 (range: 118–1567 cc) for all patients; two patients had large breast volume (1119 cc and 1567 cc, resp.). Hormone therapy was administered to 24 (60%) women. Anemia was recorded in 7 patients (17.5%); 4 of them did not show fatigue and/or depression, and 3 patients had depression and fatigue; no patient had anemia and fatigue. Breast volume, hormone therapy, and anemia were not statistically related to fatigue and/or cytokines levels (data not shown).

### 3.1. Association between Fatigue, Erythema, and Inflammatory Markers

At step 1 of the Heckman two-step correction, we found that erythema significantly influences the increase in proinflammatory cytokines levels (IL-1*β*, IL-2, IL-6, and TNF-*α*), as stated by the corresponding *P* value in (1) (*P* = 0.00001); results are shown in Table 3.

As it can be observed by looking at Table 4, reporting the estimates for the step 2 equation, fatigue symptoms are significantly influenced by increased blood levels of proinflammatory cytokines (*P* = 0.026). According to the adopted model formulation, the onset of skin erythema may lead to increased levels of proinflammatory cytokines, and these, in turn, may increase the risk for fatigue-related symptoms. Resulting from the significant effect of the inverse Mill's ratio (*M*_*it*_) in the estimated equation (2), the presence of increased proinflammatory markers could not be considered exogenous. Therefore, the suggested correction is likely to be necessary to provide reliable estimates while accounting for potential endogeneity of this variable.

## 4. Discussion 

The prevalence of fatigue has been shown to increase during treatment regarding 60–96% of the cancer-affected patients compared to about 30% at diagnosis [3, 10, 15, 24, 25]. In the present study, CRF during radiotherapy was observed in a lower fraction of patients when compared to other studies; only 17.5% of the patients developed fatigue symptoms during treatment. This may be due to the strict definition of CRF that we have used, according to Cella et al. [12, 14]. Some overlapping subjective symptoms of fatigue and depression may lead to incorrect fatigue diagnosis. Major depressive disorders or fatigue may share similar biological bases, as postulated elsewhere [6]. Thus, it is very important to distinguish CRF from major depressive disorders. Moreover, the routine use of antidepressant therapy for managing CRF was more effective in reducing depression rather than fatigue, suggesting different pathways as well as the need for different treatment interventions [7]. Also, chronic comorbid conditions are an important determinant for fatigue severity than cancer treatment among breast cancer patients [26]. For this reason, breast cancer patients with concomitant illness were excluded from our study to avoid statistical bias. Cancer-related fatigue is considered as a multidimensional symptom related to the activation of several immunomodulatory pathways due to genetic, psychological, and physiological factors [27]. Biochemical markers such as systemic inflammatory markers, specific lymphocytic subpopulations, and proinflammatory cytokines have been investigated as factors inducing CRF [2, 4–6, 8, 9, 20, 24, 27–31]. In particular, the role of proinflammatory cytokines has been largely studied with contradictory results. Several authors have described a correlation between increased levels of cytokines and fatigue, while no correlation was found by others [2–5]. Therefore, we must consider that these series of patients presented heterogeneous characteristics (gender, different neoplasms, and stage of disease), therapy (radiotherapy and/or chemotherapy and/or surgery), fatigue assessment criteria, and several other factors considered as statistical covariates. In addition, many studies referred to surviving patients. It remains unclear the reason why young women, without comorbidity, undergoing adjuvant radiotherapy for early stage breast cancer develop fatigue during therapy. In our study, a significant correlation between the occurrence of severe erythema and fatigue was noticed during RT that could be on the basis of the underlying biological mechanisms. To our knowledge, this is the first attempt to study the association between breast skin erythema, proinflammatory cytokine levels, and fatigue symptoms in women with early stage breast cancer undergoing exclusive RT.

Skin erythema is the most frequent side effect during radiotherapy but the erythema correlation with the increase of proinflammatory cytokines and the onset of fatigue has never been studied [8]. Radiation-induced erythema is due to the damage of the derma germinal layer and the DNA direct-injure causing the clonogenic death of basal epithelial cells. Moreover, a complex cascade of biological events based on innate and adaptive immune response to radiation is also involved [32–34]. Molecules released by injured cells can activate specific receptors that promote NF-*κ*B signalling and the expression of proinflammatory cytokines [28]. Although several studies have determined an association between inflammatory markers (C reactive protein, neutrophils, monocytes, and lymphocytes), cytokines levels (IL6, IL1b), and markers of cytokine activity (IL-1ra, sTNF-RII) and fatigue, no causation has been defined that support this association.

In the current study, increased levels of proinflammatory cytokines (IL-1*β*, IL-2, IL-6, and TNF-*α*) seemed to be related to the presence of high-grade breast skin erythema during radiation treatment (*P* = 0.0001) and fatigue symptoms during treatment were significantly associated with increased blood levels of these biochemical markers (*P* = 0.026).

The statistical association between fatigue and proinflammatory cytokine levels and between a cutaneous erythema and fatigue may suggest a casual link between RT-related cutaneous erythema and fatigue mediated by increased cytokine levels. Moreover, we described a typical temporal trend of the fatigue that started from the 3rd week of treatment, peaked between the 4th and the 5th week and decreased after the RT completion, with the same trend over time of the erythema appearance and the increased cytokine levels. In several studies, the IL-6 level has been related to fatigue in breast cancer patients during RT, although, this association was not confirmed by others [2, 15, 17]. Furthermore, TNF-*α*, IL2, and IL1b have not been associated with fatigue and RT in breast cancer and other malignancies [2, 27, 28].

Moreover, the RT parameters that can induce fatigue in breast cancer patients are poorly understood. Altered fractionation did not change the incidence and severity of fatigue, while the lowest irradiated volume was related to the lowest level of fatigue [35–37]. In our study, we analysed the large breast volume as a statistical parameter but no statistical association between breast volume and fatigue was found, probably due to the small cohort of patients. The current study presents limits such as the small sample size due to selection and eligibility criteria for inclusion and the small number of patients that present reliable fatigue symptoms. On the other hand, a quite complex statistical analysis has been performed to reduce spurious conclusions that may be caused by the presence of subject heterogeneity and potential endogeneity of some risk factors. This is a preliminary investigation, but the evaluation of patients with homogeneous characteristics was intended to minimize any confounding of the outcome.

## 5. Conclusions

Our study showed that fatigue symptoms seem to be related to proinflammatory cytokines that increase during radiation therapy; this increase is correlated with concurrent high-grade breast skin erythema. These results may suggest that high-grade breast skin erythema during radiotherapy course might be responsible for biological mechanisms of fatigue, activating serum proinflammatory cytokine production. A possible radiation therapy modulation or new drugs erythema-targeted can be developed to reduce skin erythema intensity and fatigue, increasing adherence to therapy and quality of life.

## Figures and Tables

**Table 1:** Patients' characteristics (*n* = 40).

	Number of patients (% of total)
Mean age (years) 55	
Range (years) 40–73	
*T*, stage	
Tis	19 (48)
T1	21 (52)
*N*, stage	
N0	40 (100)
Histological type	
DCIS	16 (40)
Ductal	17 (43)
Lobular	2 (5)
Others	5 (12)
Tumor grade (G)	
Poorly differentiated (G3)	10 (25)
Moderately differentiated (G2)	13 (32)
Well differentiated (G1)	17 (43)

Radiotherapy schedule	
50 Gy/2 Gy	19 (48)
50 Gy/2 Gy + Boost 10 Gy/2.5 Gy	21 (52)

**Table 2:** Cytokine levels at baseline compared to postradiotherapy levels (*P* values).

	Mean values of cytokines (pg/mL)					
	IL-1*β*	IL-2	IL-6	IL-8	MCP-1	TNF-*α*
HD	*0*.*106 *	*2*.*103 *	*0*.*921 *	*3*.*241 *	*350*.*54 *	*0*.*948 *
Patients pre-RT	1.9	3.05	2.65	5.309	245.52	1.41
Patients 4 weeks after RT	4.27	4.84	12.95	19.03	480.38	4.84
Patients 6 months after RT	3.49	3.58	6.34	7.83	379.97	3.58
*t*-test (HD versus pre-RT)	5.42*e* − 17*	*0*.00278*	2.44*e* − 09*	*0*.*42687 *	*0*.04959*	7.702*e* − 05*
*t*-test (pre-RT versus 4 w)	0.02682*	0.00016*	0.04853*	0.05191	0.06744	0.00165*
*t*-test (pre-RT versus 6 m)	0.09564	0.0673	0.3087	0.05585	0.24408	0.00826*

*P < 0.05.

**Table 3:** Step  1—Heckman model: parameters estimation of correlation between erythema and proinflammatory cytokines levels.

Variable	Coefficient	Standard Error	*P* value
Probit model for the inflammatory markers			
Intercept	−1.648	0.352	2.87*e* − 06
Erythema	2.065	0.468	1.01*e* − 05
*σ* ^2^_*b*_	3.792		

**Table 4:** Step  2—Heckman model: parameters estimation of correlation between fatigue and proinflammatory cytokines levels.

Variable	Coefficient	Standard Error	*P* value
Probit model for the fatigue			
Intercept	−8.574	3.460	0.0132
Breast volume	0.004	0.003	NS
Inflammatory markers	3.075	1.381	0.0260
Hormone therapy	1.975	1.923	NS
Mi	1.967	0.875	0.0246
*σ* ^2^_*u*_	6.722		

NS: not significant.
